# Blind Source Separation for Compositional Time Series

**DOI:** 10.1007/s11004-020-09869-y

**Published:** 2020-06-09

**Authors:** Klaus Nordhausen, Gregor Fischer, Peter Filzmoser

**Affiliations:** 1 CSTAT - Computational Statistics Institute of Statistics and Mathematical Methods in Economics, Vienna University of Technology, Wiedner Hauptstr. 7, 1040 Vienna, Austria; 2 CSTAT - Computational Statistics Institute of Statistics and Mathematical Methods in Economics, Vienna University of Technology, Wiedner Hauptstr. 8-10, 1040 Vienna, Austria

**Keywords:** Second-order source separation, Stochastic volatility, Nonstationary source separation, Isometric log-ratio coordinates, 62M10, 60G35, 92C55

## Abstract

Many geological phenomena are regularly measured over time to follow developments and changes. For many of these phenomena, the absolute values are not of interest, but rather the relative information, which means that the data are compositional time series. Thus, the serial nature and the compositional geometry should be considered when analyzing the data. Multivariate time series are already challenging, especially if they are higher dimensional, and latent variable models are a popular way to deal with this kind of data. Blind source separation techniques are well-established latent factor models for time series, with many variants covering quite different time series models. Here, several such methods and their assumptions are reviewed, and it is shown how they can be applied to high-dimensional compositional time series. Also, a novel blind source separation method is suggested which is quite flexible regarding the assumptions of the latent time series. The methodology is illustrated using simulations and in an application to light absorbance data from water samples taken from a small stream in Lower Austria.

## Introduction

There is an increasing awareness that many multivariate data sets are of a compositional nature. This means that the main interest is not in the absolute values as they are reported, but in relative information, for instance in terms of (log-)ratios of the values between the variables. In that way, the total sum of an observation is no longer important, and in many applications, the total is just an arbitrary value used for normalizing the data, such as 1 or 100%. When working with (log-)ratios, such a total sum normalization would also not change the (log-)ratio, and hence the analysis is independent of a normalization. Thus, for instance, the analysis of employment data reported from different sectors would be the same if they where treated in absolute numbers or as percentages. Textbooks about compositional data are, for example, Aitchison (2003) or Filzmoser et al. (2018) which give a general treatment of such data. The same problem occurs naturally when such compositions are observed over time. Compositional time series (CTS) analysis is a topic which has begun receiving more attention recently, see for example Bergman and Holmquist (2014), Dawson et al. (2014), Kynclova et al. (2015), Snyder et al. (2017), AL-Dhurafi et al. (2018).

In a recent CTS review paper, Larrosa (2017) gives a general overview and mentions as example applications socioeconomic time series, industrial production time series, polls data and epidemiologic time series. As Larrosa (2017) mentions, the original CTS must first be expressed in the usual Euclidean geometry, before standard time series methods can be applied. It is also pointed out that in the literature, more attention is paid to CTS from the Bayesian point of view.

Compositional data are by definition multivariate, and fitting sophisticated time series models in more than three dimensions can be quite demanding, as pointed out, for example, in Chang et al. (2018). Therefore, it is tempting to assume latent components which allow individual univariate modeling. This is the approach followed in this paper. The idea is to first represent the CTS in the Euclidean space, and then assume in that space a blind source separation (BSS) model. Different ways to approach the estimation of the latent components in BSS will be outlined, and how to yield then again the multivariate CTS in the original space.

The structure of the paper is as follows. Section 2 provides general details of compositional data analysis, and explains how the data can be presented in the standard Euclidean geometry. In Sect. 3, several BSS methods for time series are reviewed; they are based on different assumptions of the underlying stochastic processes. Also, a new BSS method is suggested which is compared to the existing ones in a simulation study. Section 4 gives a recommendation of how to perform BSS for compositional time series. The methodology is illustrated in Sect. 5 with an example where light absorption data from water samples in a small stream in Lower Austria are observed over time. Finally, general conclusions are provided in Sect. 6.

## Compositional Time Series and Coordinate Representations

Let $\mathbf{x}_t = (x_{1,t},\ldots , x_{p,t})^\top$ be a compositional time series, observed at time points $t=1,\ldots ,T$. For the analysis of compositional data, the interest is not in the absolute information expressed directly by the values in $\mathbf{x}_t$, but rather in the relative information in terms of the (log-)ratios between the components of $\mathbf{x}_t$ (Filzmoser et al. 2018). For example, considering all pairwise log-ratios with $x_{1,t}$ results in

$$\begin{aligned} \ln \frac{x_{1,t}}{x_{1,t}}=0, \ln \frac{x_{1,t}}{x_{2,t}} ,\ldots ,\ln \frac{x_{1,t}}{x_{p,t}}, \end{aligned}$$

and they can be aggregated as

1 $$\begin{aligned} x_{1,t}^{\mathrm{clr}}=\frac{1}{p}\left( \ln \frac{x_{1,t}}{x_{2,t}} +\cdots +\ln \frac{x_{1,t}}{x_{p,t}} \right) = \ln \frac{x_{1,t}}{\root p \of {\prod _{i=1}^px_{i,t}}} , \end{aligned}$$

with the geometric mean in the denominator. $x_{1,t}^{\mathrm{clr}}$ is called the centered log-ratio (clr) coefficient for $x_{1,t}$, and similarly one can also express the other components in terms of clr coefficients (Aitchison 2003)

2 $$\begin{aligned} \mathbf{x}_t^{\mathrm{clr}}=(x_{1,t}^{\mathrm{clr}}, \ldots ,x_{p,t}^{\mathrm{clr}})^\top = \left( \ln \frac{x_{1,t}}{\root p \of {\prod _{i=1}^px_{i,t}}}, \ldots , \ln \frac{x_{p,t}}{\root p \of {\prod _{i=1}^px_{i,t}}}\right) ^\top . \end{aligned}$$

Thus, $\mathbf{x}_t^{\mathrm{clr}}$ contains all relative information in terms of aggregated pairwise log-ratios. It is not difficult to see that $x_{1,t}^{\mathrm{clr}}+ \cdots +x_{p,t}^{\mathrm{clr}}=0$, and thus it can be inconvenient to work with clr coefficients if full-rank matrices are necessary. As a way out, isometric log-ratio (ilr) coordinates have been proposed, which refer to a family of coordinates building an orthonormal basis in the ($p-1$)-dimensional hyperplane formed by clr coefficients (Egozcue et al. 2003). Among the infinitely many possibilities to define such an orthonormal basis system, one particular choice are so-called pivot coordinates, defined as (Filzmoser et al. 2018)

3 $$\begin{aligned} x_{i,t}^{\mathrm{ilr}}=\sqrt{\frac{p-i}{p-i+1}}\,\ln \frac{x_{i,t}}{\root p-i \of {\prod _{j=i+1}^p x_{j,t}}} \quad \text{ for } \quad i=1,\ldots ,p-1 , \end{aligned}$$

with the inverse relationship

$$\begin{aligned} x_{1,t}= & {} \exp \left( \sqrt{\frac{p-1}{p}} x_{1,t}^{\mathrm{ilr}} \right) ,\\ x_{i,t}= & {} \exp \left( - \sum _{j=1}^{i-1} \frac{x_{j,t}^{\mathrm{ilr}}}{\sqrt{(p-j+1)(p-j)}} + \sqrt{\frac{p-i}{p-i+1}} x_{i,t}^{\mathrm{ilr}}\right) , \quad i=2,\ldots ,p-1,\\ x_{p,t}= & {} \exp \left( - \sum _{j=1}^{p-1} \frac{x_{j,t}^{\mathrm{ilr}}}{\sqrt{(p-j+1)(p-j)}} \right) . \end{aligned}$$

Since $x_{1,t}^{\mathrm{ilr}}=\sqrt{\frac{p}{p-1}}x_{1,t}^{\mathrm{clr}}$, this coordinate system allows for a specific interpretation of the first component, because it summarizes all relative information about $x_{1,t}$ in terms of averaged log-ratios.

In fact, the sample space of compositional data $\mathbf{x}_t$ is the *p*-dimensional simplex (Aitchison 2003), and ilr coordinates $\mathbf{x}_t^{\mathrm{ilr}}=\left( x_{1,t}^{\mathrm{ilr}},\ldots ,x_{p-1,t}^{\mathrm{ilr}}\right) ^\top$ are one possibility for expressing the compositions in the $(p-1)$-dimensional real Euclidean space. More details on geometrical properties are provided, for example, in Pawlowsky-Glahn et al. (2015). Moreover, different (orthonormal) ilr coordinate systems are just orthonormal rotations of each other, so they are obtained by a multiplication e.g. of $\mathbf{x}_t^{\mathrm{ilr}}$ with an orthonormal matrix of dimension $p-1$ (Egozcue et al. 2003). It will be important later on to show that the results of CTS analysis are invariant with respect to the specific choice of the ilr coordinates. Finally, there is also a link between clr coefficients and the ilr coordinates from Eq. (3),

4 $$\begin{aligned} \mathbf{x}_t^{\mathrm{clr}} = \mathbf{V} \mathbf{x}_t^{\mathrm{ilr}} \quad \text{ and } \quad \mathbf{x}_t^{\mathrm{ilr}} = \mathbf{V}^\top \mathbf{x}_t^{\mathrm{clr}} , \end{aligned}$$

with a matrix $\mathbf{V}$ of dimension $p\times (p-1)$, with columns

5 $$\begin{aligned} \mathbf{v}_j =\sqrt{\frac{p-j}{p-j+1}}\left( 0,\ldots ,0,1,-\frac{1}{p-j},\ldots ,-\frac{1}{p-j}\right) ^\top , \end{aligned}$$

for $j=1,\ldots ,p-1$, with $j-1$ zero entries (Egozcue et al. 2003). Of course, a similar relationship holds for other choices of ilr coordinates.

## Blind Source Separation for Time Series

Blind source separation (BSS) is a popular multivariate approach for decomposing multivariate data into uncorrelated components which are useful for dimension reduction, and intended for an easier interpretation or easier modeling of the data (for overviews see, for example, Comon and Jutten 2010; Nordhausen and Oja 2018). BSS is quite popular for biomedical signal analysis or financial time series decomposition, and in many other fields as well.

The basic BSS model assumes that the observable *p*-variate time series $\mathbf{x}=(\mathbf{x}_t)_{t=0,\pm 1,\pm 2, \ldots }$ satisfies

$$\begin{aligned} \mathbf{x}_t=\varvec{\mu }+\varvec{\varOmega }\mathbf{z}_t,\quad t=0,\pm 1,\pm 2,\ldots , \end{aligned}$$

where $\varvec{\mu }\in {\mathbb {R}}^p$ is a *p*-variate location vector, $\varvec{\varOmega }\in {\mathbb {R}}^{p\times p}$ is a full-rank mixing matrix and $\mathbf{z}=(\mathbf{z}_t)_{t=0,\pm 1,\pm 2, \ldots }$ is a *p*-variate latent time series having $E(\mathbf{z}_t)=\mathbf 0$. The goal in BSS is to estimate $\mathbf{z}_t$. The location vector $\varvec{\mu }$ is usually considered a nuisance parameter, which is set in the following to zero for simplicity.

Clearly, $\mathbf{z}_t$ cannot be estimated without further assumptions, and there are different BSS methods which differ in the assumptions that are made. The most popular BSS method is independent component analysis (ICA), which assumes that $\mathbf{z}_t$ has independent non-Gaussian components. However, most ICA methods are developed for iid data and are therefore not of interest in this paper. Here, the focus is on BSS approaches specific for time series which make use of the serial information in the data.

### Second-Order Source Separation

The most established BSS approach for time series assumes the second-order source separation (SOS) model, where the sources are *p*-variate time series $\mathbf{z}=(\mathbf{z}_t)_{t=0,\pm 1,\pm 2,\dots }$ which satisfy the assumptions

1. $$E(\mathbf{z}_t)= \mathbf 0$$ and $$E(\mathbf{z}_t^\top \mathbf{z}_t)= \mathbf{I}_p$$, and
2. $E(\mathbf{z}_t \mathbf{z}_{t+\tau }^\top )= \mathbf{D}_\tau$ is diagonal for all $\tau =1,2,\ldots$

These two assumptions imply that the components of $\mathbf{z}$ are weakly stationary and uncorrelated time series where, however, often for convenience, the stronger assumption of independence is made. In the following, two classical SOS methods are described.

#### Definition 1

The AMUSE [algorithm for multiple unknown signals extraction (Tong et al. 1990)] method simultaneously diagonalizes the following two matrices

$$\begin{aligned} \mathbf{COV} (\mathbf{x}) \ \ \text{ and } \ \ \mathbf{S}_\tau (\mathbf{x})= E[(\mathbf{x}_t- E(\mathbf{x}_t)) (\mathbf{x}_{t+\tau }-E(\mathbf{x}_t))^\top ], \end{aligned}$$

where $\mathbf{COV}$ denotes the regular covariance matrix and $\mathbf{S}_\tau$ the autocovariance matrix at lag $\tau$. The AMUSE unmixing matrix $\varvec{\varGamma }_\tau$ then satisfies

$$\begin{aligned} \varvec{\varGamma }_\tau \mathbf{COV} (\mathbf{x})\varvec{\varGamma }_\tau ^\top = \mathbf{I}_p \ \ \text{ and } \ \ \varvec{\varGamma }_\tau \mathbf{S}_\tau (\mathbf{x})\varvec{\varGamma }_\tau ^\top = \mathbf{D}_\tau , \end{aligned}$$

where $\mathbf{D}_\tau$ is a diagonal matrix with decreasing diagonal elements.

AMUSE can therefore be solved via a generalized eigenvector decomposition, and thus $\varvec{\varGamma }_\tau$ contains the eigenvectors of $\mathbf{COV} (\mathbf{x})^{-1} \mathbf{S}_\tau (\mathbf{x})$, and $\mathbf{D}_\tau$ the corresponding eigenvalues. This also means that in order to work, AMUSE requires distinct eigenvalues, which implies that the autocorrelations with the chosen lag need to be different for the source components. Statistical properties of AMUSE are given, for example, in Miettinen et al. (2012), and it is well known that the choice of the lag has a huge impact on the performance of AMUSE.

Belouchrani et al. (1997) suggested avoiding this dependency by not only diagonalizing two matrices, but adding more autocovariance matrices with different lags.

#### Definition 2

The SOBI (second-order blind identification) method first whitens the observable time series using the covariance matrix $\mathbf{COV} (\mathbf{x})$. Then *K* autocovariance matrices for a set of distinct lags ${\mathcal {T}}=\{\tau _1,\dots ,\tau _K\}$ are computed for the whitened time series $\mathbf{x}_t^{st}$, yielding

$$\begin{aligned} \mathbf{S}_{\tau _1}(\mathbf{x}_t^{st}), \ldots , \mathbf{S}_{\tau _K}(\mathbf{x}_t^{st}). \end{aligned}$$

The SOBI unmixing matrix $\varvec{\varGamma }_{\mathcal {T}}$ is given by

$$\begin{aligned} \varvec{\varGamma }_{\mathcal {T}} = \mathbf{U} \mathbf{COV} (\mathbf{x}_t)^{-1/2}, \end{aligned}$$

where the orthogonal $p \times p$ matrix $\mathbf{U}=(\mathbf{u}_1,\ldots ,\mathbf{u}_p)^\top$ maximizes

$$\begin{aligned} \sum _{\tau \in {\mathcal {T}}} \sum _{i=1}^p (\mathbf{u}_i^\top \mathbf{S}_\tau (\mathbf{x}_t^{st})\mathbf{u}_i)^2. \end{aligned}$$

Hence, SOBI can be seen as a method that first whitens the data and tries to make the *K* autocovariance matrices as diagonal as possible. Many algorithms are available to solve this problem which, however, give estimates with different statistical properties. Some algorithms and the properties of the resulting estimators are discussed, for example, in Miettinen et al. (2014), Illner et al. (2015), Miettinen et al. (2016). In general, it is considered that in an SOS framework, SOBI is preferable over AMUSE.

SOS methods exploit second-order properties of the stochastic processes and therefore assume that the components have nontrivial autocovariance matrices which differ in at least one lag. This means that SOS is working well when, for example, autoregressive-moving-average (ARMA) modeling is natural. However, for stochastic volatility processes which are popular in financial applications, for instance, SOS fails, as there is no second-order information.

### BSS for Time Series with Stochastic Volatility

In order to deal with stochastic volatility time series, Matilainen et al. (2015) considered the stochastic volatility independent component model for time series models, which will be denoted by SV. In the SV model it is assumed that

1. $$E(\mathbf{z}_t)=\mathbf 0$$ and $$E(\mathbf{z}_t \mathbf{z}_t^\top )= \mathbf{I}_p$$, and
2. each component of $\mathbf{z}_t$ exhibits stochastic volatility features and has finite fourth moments and cross-moments where no two components are identical at all lags.

In the following, three methods will be introduced to estimate the sources in SV, namely gFOBI, gJADE and vSOBI.

#### Definition 3

gFOBI (Matilainen et al. 2015) first whitens the time series to yield the time series $\mathbf{x}_{t}^{st}$ and subsequently finds an orthogonal matrix $\mathbf{U}\in {\mathbb {R}}^{p\times p}$ such that it maximizes for a set of lags ${\mathcal {T}}=\{\tau _1,\ldots ,\tau _K\}$

$$\begin{aligned} \sum _{\tau \in {\mathcal {T}}}\sum _{i=1}^p(\mathbf{u}^\top _i\mathbf{B}_\tau (\mathbf{x}_t^{st})\mathbf{u}_i)^2, \end{aligned}$$

where $\mathbf{B}_\tau (\mathbf{x}_t^{st})=E[\mathbf{x}_{t+\tau }^{st}{\mathbf{x}^{st}}^\top _t\mathbf{x}_t^{st}{\mathbf{x}^{st}_{t+\tau }}^\top ]$ is the matrix of fourth-order cross-moments at lag $\tau$. The gFOBI unmixing matrix is then given by $\mathbf \varGamma _{\mathcal {T}}=\mathbf{U} \mathbf{COV} (\mathbf{x}_t)^{-1/2}$.

For gJADE, the cross-cumulant matrices at lag $\tau$ are defined as

$$\begin{aligned} \mathbf{C}^{jk}_\tau (\mathbf{x}_t)=E[\mathbf{x}_{t+\tau }\mathbf{x}_t^\top \mathbf{E}^{jk}\mathbf{x}_t\mathbf{x}_{t+\tau }^\top ]-\mathbf{S}_\tau (\mathbf{x}_t)(\mathbf{E}^{jk}+\mathbf{E}^{kj})\mathbf{S}_\tau (\mathbf{x}_t)^\top -\mathrm {trace}(\mathbf{E}^{jk}) \mathbf{I}_p, \end{aligned}$$

where $\mathbf{E}^{jk}=\mathbf{e}_j\mathbf{e}^\top _k$, $j,k=1,\dots ,p$ with $\mathbf{e}_i$ denotes a unit vector with 1 at entry *i*.

#### Definition 4

gJADE (Matilainen et al. 2015) again first whitens the time series and then searches the orthogonal matrix $\mathbf{U}\in {\mathbb {R}}^{p\times p}$ which maximizes

$$\begin{aligned} \sum _{\tau \in {\mathcal {T}}}\sum _{i=1}^p\sum _{j=1}^p\sum _{k=1}^p(\mathbf{u}^\top _i\mathbf{C}^{jk}_\tau (\mathbf{x}_t^{st})\mathbf{u}_i)^2, \end{aligned}$$

given the set of lags ${\mathcal {T}}=\{\tau _1,\ldots ,\tau _K\}$. Accordingly, the gJADE unmixing matrix is given by $\mathbf \varGamma _{\mathcal {T}}=\mathbf{U} \mathbf{COV} (\mathbf{x}_t)^{-1/2}$.

Both methods, gFOBI and gJADE, are generalizations of the iid ICA method FOBI [fourth-order blind identification (Cardoso 1989)] and JADE [joint approximate diagonalization of eigenmatrices (Cardoso and Souloumiac 1993)] and are here obtained simply by setting ${\mathcal {T}}=\{0\}$.

A variant of SOBI for the SV case was proposed in Matilainen et al. (2017).

#### Definition 5

vSOBI uses the covariance matrix to whiten the time series, and for a fixed set of lags ${\mathcal {T}}=\{\tau _1,\ldots ,\tau _K\}$ it finds the orthogonal matrix $\mathbf{U}\in {\mathbb {R}}^{p\times p}$ which maximizes

$$\begin{aligned} \sum _{\tau \in {\mathcal {T}}}\sum _{i=1}^p (E[G(\mathbf{u}^\top _i\mathbf{x}^{st}_t)G(\mathbf{u}^\top _i\mathbf{x}^{st}_{t+\tau })]-E[G(\mathbf{u}^\top _i\mathbf{x}^{st}_t)]^2)^2, \end{aligned}$$

where *G* can be any twice continuously differentiable function. The vSOBI unmixing matrix is then $\varvec{\varGamma }_{\mathcal {T}}=\mathbf{U} \mathbf{COV} (\mathbf{x}_t)^{-1/2}$.

Popular choices for *G* are $G(y)=y^2$ and $G(y)=\log (\cosh (y))$. For comparisons of gFOBI, gJADE and vSOBI, see Matilainen et al. (2017).

Both the SOS model and the SV model assume stationarity of the time series. There is, however, also a BSS model for nonstationary time series.

### Nonstationary Source Separation

The nonstationary source separation (NSS) relaxes the assumption of stationarity.

The NSS model makes the following assumptions:

1. $$E(\mathbf{z}_t)= \mathbf 0$$ for all *t*,
2. $E(\mathbf{z}_t \mathbf{z}_t^\top )$ is positive definite and diagonal for all *t*,
3. $E(\mathbf{z}_t \mathbf{z}_{t+\tau }^\top )$ is diagonal for all *t* and $\tau$.

Again, (NSS2) implies only uncorrelatedness, but in practice often independence is assumed. Hence, in the NSS model, the source components are uncorrelated/independent and they have a constant mean. However, the variances are allowed to change over time as well as the autocovariance matrices. A special case here is, for example, a block-stationary model, where the time series can be divided into blocks so that an SOS model holds for each block.

Three approaches for NSS are considered here, namely NSS-SD, NSS-JD and NSS-TD-JD (Choi and Cichocki 2000a, b), which are methods that take the nonstationarity of the variances into account. The third method is specifically intended for a block-stationary model. For the description of all three methods, the following local scatter matrices are required

$$\begin{aligned} \mathbf{S}_{T,\tau }(\mathbf{x})=\frac{1}{|T|-\tau }\sum _{t\in T} E [(\mathbf{x}_t- E(\mathbf{x}_t)) (\mathbf{x}_{t+\tau }- E(\mathbf{x}_t))^\top ], \end{aligned}$$

where *T* is a finite time interval and $\tau \in \{0,1,\ldots \}$.

#### Definition 6

The NSS-SD unmixing matrix simultaneously diagonalizes $\mathbf{S}_{T_1,0}(F_x)$ and $\mathbf{S}_{T_2,0}(F_x)$, where $T_1,\ T_2$ are separate time intervals. $T_1$ and $T_2$ should be chosen so that $\mathbf{S}_{T_1,0}(\mathbf{x})$ and $\mathbf{S}_{T_2,0}(\mathbf{x})$ are as different as possible. As with AMUSE, it is obtained via a generalized eigenvector decomposition.

NSS-SD suffers from the same drawback as AMUSE: the choice of how to divide the time range into the two intervals has a great impact on the separation performance. In order to depend less on this choice, NSS-JD divides the time range into more than two intervals.

#### Definition 7

NSS-JD whitens the time series using the covariance matrix $\mathbf{S}_{[1,n],0}(\mathbf{x})$ computed from all the observations. The time range is then divided into *K* nonoverlapping intervals $T= \{T_1,\ldots ,T_K\}$. The NSS-JD unmixing matrix is then $\varvec{\varGamma }_T=\mathbf{U} \mathbf{S}_{[1,n],0}(\mathbf{x})^{-1/2}$, where the orthogonal matrix $\mathbf{U}\in {\mathbb {R}}^{p\times p}$ maximizes

$$\begin{aligned} \sum _{T}\sum _{i=1}^p(\mathbf{u}^\top _i\mathbf{S}_{T_j,0}(\mathbf{x}_t^{st})\mathbf{u}_i)^2. \end{aligned}$$

Both NSS-SD and NSS-JD ignore serial dependence but can also be applied when the observations are not equidistant. If, however, the SOS model holds approximately within the interval, information coming from the autocovariance matrices within the intervals can be exploited as well, as done by the NSS-TD-JD method.

#### Definition 8

NSS-JD-TD again whitens the time series using the covariance matrix $\mathbf{S}_{[1,n],0}(\mathbf{x})$. The time range is again divided into *K* nonoverlapping intervals $T= \{T_1,\ldots ,T_K\}$, and a set of *L* lags ${\mathcal {T}}=\{\tau _1,\ldots ,\tau _L\}$ is chosen. The NSS-JD-TD unmixing matrix is hence $\varvec{\varGamma }_{T,{\mathcal {T}}}=\mathbf{U} \mathbf{S}_{[1,n],0}(\mathbf{x})^{-1/2}$, where the orthogonal matrix $\mathbf{U}\in {\mathbb {R}}^{p\times p}$ maximizes

$$\begin{aligned} \sum _{T} \sum _{{\mathcal {T}}}\sum _{i=1}^p(\mathbf{u}^\top _i\mathbf{S}_{T_k,\tau _j}(\mathbf{x}_t^{st})\mathbf{u}_i)^2. \end{aligned}$$

### Common Properties of all the BSS Methods

All the BSS methods mentioned above specify a specific BSS model by making different additional assumptions about the latent components. Note, however, that all these BSS models are ill-defined. In all models, the signs of the components and their order are not fixed. In addition, in the NSS model, the scales of the components are also not fixed. However, in practical applications this is usually not a problem, and it should be considered just when comparing different estimators.

A property of all the above mentioned methods is that they are affine equivariant in the sense that

$$\begin{aligned} \varvec{\varGamma }(\mathbf{x}) \mathbf{x} = \mathbf{J} \mathbf{P} \varvec{\varGamma }(\mathbf{x}^*) \mathbf{x}^*, \end{aligned}$$

where $\mathbf{x}^* = \mathbf{A} \mathbf{x}$ for any full-rank matrix $\mathbf{A} \in {\mathbb {R}}^{p \times p}$, where $\mathbf{J}$ is a sign change matrix (a diagonal matrix with $\pm 1$ on the diagonal) and $\mathbf{P}$ a permutation matrix. This means that the mixing matrix has no impact on the performance of each method, and at most the order and the signs of the components can change. This also holds if $\mathbf{x}$ does not follow a BSS model.

This affine equivariance property will be important later when BSS is applied in the CTS context.

### A New BSS Method

Basically, for all BSS methods above (except vSOBI), the defined matrices are diagonal for the latent components, and the unmixing matrix is found as the matrix which jointly diagonalizes two or more such matrices. Depending on which kind of generating processes are assumed for the latent components, the appropriate BSS method should be chosen. Often this choice is based on subject matter knowledge and visual inspection.

A novel approach is suggested here, a new combination of the methods above, which is inspired by NSS-JD-TD. NSS-JD-TD basically combines the SOS model and the NSS model but ignores the SV model. Therefore, the idea is to also include in the joint diagonalization process matrices targeting the SV model. The suggestion is therefore to also include the matrices defined for gFOBI.

The new BSS method is thus defined as:

#### Definition 9

NSS-SOBI-gFOBI again whitens the time series using the covariance matrix $\mathbf{S}_{[1,n],0}(\mathbf{x})$. The time range is afterwards divided into *K* nonoverlapping intervals $T= \{T_1,\ldots ,T_K\}$, and two sets of $L_1$ and $L_2$ lags ${\mathcal {T}}_1=\{\tau _{1,1},\ldots ,\tau _{1,L_1}\}$ and ${\mathcal {T}}_2 =\{\tau _{2,1},\ldots ,\tau _{ 2, L_2}\}$ are chosen and combined as

$$\begin{aligned} {\mathcal {T}}^* = \{\tau _{1,1},\ldots ,\tau _{1,L_1},\tau _{2,1},\ldots ,\tau _{ 2, L_2}\} = \{\tau ^*_1,\ldots ,\tau ^*_{L_1+L_2}\}. \end{aligned}$$

Define for a given interval $T_i$ the $L_1+L_2$ matrices

$$\begin{aligned} \mathbf{V}_{T_i,j} = \left\{ \begin{array}{ll} \mathbf{S}_{T_i,\tau ^*_j}(\mathbf{x}_t^{st}) &{} \text{ for } \ j \in 1,\ldots , L_1\\ \mathbf{B}_{T_i,\tau ^*_j}(\mathbf{x}_t^{st}) &{} \text{ for } \ j \in L_1+1,\ldots , L_1 + L_2 .\\ \end{array} \right. \end{aligned}$$

The matrix $\mathbf{B}_{T_i,\tau ^*_j}(\mathbf{x}_t^{st})$ is the fourth-order cross-moments matrix at lag $\tau ^*_j$ for time interval $T_i$.

The NSS-SOBI-gFOBI unmixing matrix is defined as $\varvec{\varGamma }_{T,{\mathcal {T}}^*}=\mathbf{U} \mathbf{S}_{[1,n],0}(\mathbf{x})^{-1/2}$, where the orthogonal matrix $\mathbf{U}\in {\mathbb {R}}^{p\times p}$ maximizes

$$\begin{aligned} \sum _{T} \sum _{{\mathcal {T}}^*}\sum _{i=1}^p(\mathbf{u}^\top _i\mathbf \alpha _j V_{T_k,\tau _j^*}(\mathbf{x}_t^{st})\mathbf{u}_i)^2. \end{aligned}$$

where $\alpha _j$ is the weight for matrix $V_{T_k,\tau _j^*}$.

Note that for all previous methods, the matrices to be jointly diagonalized come from the same family, and therefore they were directly comparable and it was natural to give them all the same weight. Now, however, the matrices $\mathbf{S}_{T_i,\tau ^*_j}$ and $\mathbf{B}_{T_i,\tau ^*_j}$ are of a different nature, and it can be assumed that choosing $\alpha _j = 1$ for all *j* gives more weight to the information contained in the $\mathbf{B}$ matrices. Often for $\mathbf{B}_{0}$, which is known as the scatter matrix of fourth moments, $\alpha = 1/(p+2)$ is chosen, as then the scatter matrix is a consistent estimate for the covariance matrix at a multivariate normal model (Nordhausen et al. 2011). Another option is to use $\alpha _j = 1/\max |{V_j}{[k,l]}|$ if $\max |{V_j}{[k,l]}| > 1$, and otherwise $\alpha _j=1$. The motivation here is that there might be zero matrices which should not be up-weighted, but at the same time matrices with larger entries should be down-weighted a bit. Here, $\max |{V_j}{[k,l]}|$ denotes the element of matrix $\mathbf{V}_j$ with the largest absolute value.

## BSS for CTS

CTS are by nature multivariate processes, which makes them challenging to model. BSS methods are a convenient tool for decomposing multivariate processes into uncorrelated or independent processes, which then allows dimension reduction and univariate modeling. For BSS, however, full-rank data are required, making it necessary to express the CTS in isometric log-ratio coordinates. BSS for CTS consists of the following steps:

1. Represent $$\mathbf{x}_t$$ by $$\mathbf{x}_t^{\mathrm{ilr}}$$.
2. Apply the BSS method of interest to $\mathbf{x}_t^{\mathrm{ilr}}$, yielding the latent uncorrelated / independent processes $\mathbf{z}_t = \hat{\varvec{\varGamma }} \mathbf{x}_t^{\mathrm{ilr}}$.
3. Use $\mathbf{z}_t$ or components of it for modeling, prediction or for whatever the purpose of the analysis is.
4. If needed, re-express for example the predictions in the ilr space using $\hat{\varvec{\varGamma }}^{-1}$, and in the original sample space by inverting the inverse ilr mapping.

If, for example, interpretations of the latent series in $\mathbf{z}_t$ are required, this would often be easier when referring to the clr coefficients. Therefore, one can exploit the relationship between ilr and clr and interpret $\varvec{\varGamma }\mathbf{V}^\top$ as loading matrix.

Also note that as all the BSS methods discussed in this paper are affine equivariant, the choice of the ilr basis does not matter, and if another basis is preferred, the independent components would at most change in their order and their signs, which is not of practical relevance.

Recall that the assumptions on the BSS components are made in the ilr space, and accordingly the appropriate BSS method should be chosen. For example, for geochemical time series such as the composition of gas, water or sediments, such assumptions are difficult to make, and therefore it is recommended that general methods be used in order to be on the safe side. This will be demonstrated in the following simulation study, comparing some of the previous BSS approaches in different settings.

In the simulation study, the three different weights for NSS-SOBI-gFOBI are demonstrated and compared with the individual estimators. Thus, SOBI is used with ${\mathcal {T}}=\{1,\ldots ,6\}$, gFOBI with ${\mathcal {T}}=\{0,\ldots ,6\}$, and NSS-JD with $K=6$ and then NSS-SOBI-gFOBI with $K=6$, ${\mathcal {T}}_1=\{0,\ldots ,6\}$, ${\mathcal {T}}_2=\{0,\ldots ,6\}$, and then three choices for selecting the weights – in NSS-SOBI-gFOBI-1 all matrices get the same weight; in NSS-SOBI-gFOBI-2 all $\mathbf{B}$ matrices are divided by $p+2$, and in NSS-SOBI-gFOBI-3 they are standardized as described so that the maximal elements of matrices are below 1 or above $-1$.

Three different scenarios for the latent ilr components are considered:

LP: All *p* ilr components are linear processes.
SV: All *p* ilr components are processes with stochastic volatility.
LP & SV: *p*/2 of the ilr components are linear processes and the other half are stochastic volatility processes.

**Fig. 1:**
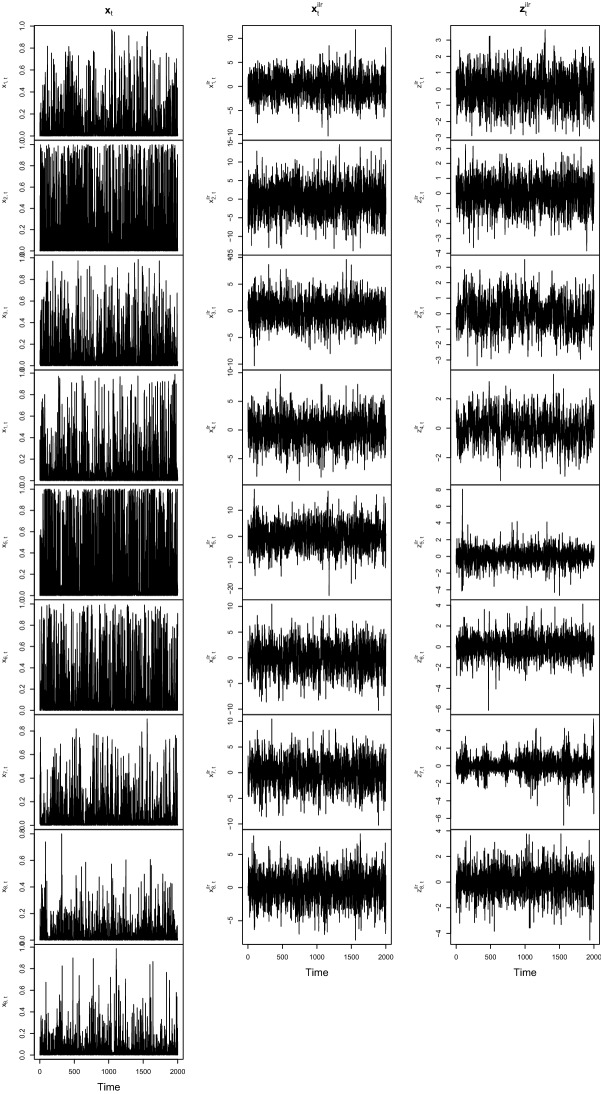
Example times series from Setting 3 with a random mixing matrix with $T=2000$

For each scenario, three different settings are considered. Both Setting 1 and Setting 2 have $p=4$, and the difference is that in Setting 1 all innovations are Gaussian, and in Setting 2 the innovations all have heavy tails coming from a logistic distribution having finite fourth moments. In Setting 3, the innovations are Gaussian and $p=8$. In all settings, due to the affine equivariance of all the methods, the mixing matrix is set without loss of generality to $\varvec{\varOmega }= \mathbf{I}_p$. For visualization purposes, however, Fig. 1 presents an example of Setting 3 for $T=2000$, where the independent components in the ilr space are shown when mixed with a matrix containing random *N*(0, 1) elements and then the resulting $p+1$-dimensional CTS.

It can be seen from Fig. 1 that it is quite difficult to guess the nature of the underlying processes when considering $\mathbf{x}_t$ or $\mathbf{x}_t^\mathrm {ilr}$. The latent components $\mathbf{z}_t^\mathrm {ilr}$ often show a clearer structure, but still it would be difficult to say what would be the best BSS method to choose. This motivates the use of methods which are valid in many different scenarios.

Recall that in a pure LP scenario, the autocovariance matrices contain the most information, whereas in an SV scenario they are all close to zero, and the matrices with the fourth cross-moments are the informative ones.

**Table 1:** Average MD index values in Setting 1 for sample sizes 1,000, 2,000 and 10,000 based on 2,000 repetitions. COMB-1, COMB-2 and COMB-3 denote NSS-SOBI-gFOBI-1, NSS-SOBI-gFOBI-2 and NSS-SOBI-gFOBI-3, respectively

Method	LP			SV			SV & LP		
	1,000	2,000	10,000	1,000	2,000	10,000	1,000	2,000	10,000
SOBI	0.306	0.273	0.150	0.589	0.571	0.539	0.231	0.205	0.177
gFOBI	0.589	0.500	0.306	0.289	0.205	0.091	0.330	0.267	0.116
NSS.JD	0.679	0.675	0.680	0.232	0.201	0.170	0.388	0.367	0.349
COMB-1	0.651	0.613	0.419	0.255	0.194	0.095	0.363	0.297	0.154
COMB-2	0.371	0.333	0.263	0.249	0.190	0.095	0.190	0.130	0.061
COMB-3	0.396	0.346	0.275	0.248	0.186	0.090	0.181	0.120	0.054

**Table 2:** Average MD index values in Setting 2 for sample sizes 1,000, 2,000 and 10,000 based on 2,000 repetitions. COMB-1, COMB-2 and COMB-3 denote NSS-SOBI-gFOBI-1, NSS-SOBI-gFOBI-2 and NSS-SOBI-gFOBI-3, respectively

Method	LP			SV			SV & LP		
	1,000	2,000	10,000	1,000	2,000	10,000	1,000	2,000	10,000
SOBI	0.312	0.279	0.148	0.565	0.532	0.460	0.213	0.188	0.139
gFOBI	0.505	0.413	0.226	0.230	0.178	0.088	0.276	0.240	0.129
NSS.JD	0.617	0.608	0.607	0.179	0.151	0.106	0.312	0.296	0.267
COMB-1	0.579	0.523	0.332	0.187	0.148	0.077	0.283	0.244	0.142
COMB-2	0.339	0.280	0.213	0.185	0.147	0.077	0.180	0.127	0.060
COMB-3	0.356	0.293	0.220	0.174	0.134	0.069	0.156	0.106	0.047

**Table 3:** Average MD index values in Setting 3 for sample sizes 1000, 2000 and 10,000 based on 2000 repetitions. COMB-1, COMB-2 and COMB-3 denote NSS-SOBI-gFOBI-1, NSS-SOBI-gFOBI-2 and NSS-SOBI-gFOBI-3, respectively

Method	LP			SV			LP & SV		
	1,000	2,000	10,000	1,000	2,000	10,000	1,000	2,000	10,000
SOBI	0.341	0.266	0.134	0.759	0.752	0.738	0.419	0.390	0.352
gFOBI	0.778	0.735	0.488	0.511	0.399	0.208	0.563	0.471	0.274
NSS.JD	0.799	0.798	0.799	0.405	0.378	0.339	0.592	0.576	0.556
COMB-1	0.800	0.794	0.753	0.464	0.399	0.237	0.597	0.547	0.397
COMB-2	0.609	0.467	0.232	0.456	0.393	0.238	0.363	0.251	0.100
COMB-3	0.614	0.481	0.242	0.471	0.408	0.265	0.334	0.232	0.093

As a performance measure in the simulation study, the minimum distance index (MD) (Ilmonen et al. 2010) is used, which is defined as

$$\begin{aligned} \mathrm{MD}(\hat{\varvec{\varGamma }}, \varvec{\varOmega }) = \frac{1}{\sqrt{p - 1}} \underset{\mathbf {C} \in {\mathcal {C}}}{\inf }\left\| \mathbf {C} \hat{\varvec{\varGamma }} \varvec{\varOmega }- \mathbf{I} _{p} \right\| , \end{aligned}$$

where $\hat{\varvec{\varGamma }}$ is the estimated unmixing matrix, $\varvec{\varOmega }$ the true mixing matrix, and ${\mathcal {C}}$ is the set of matrices all having in each row and column exactly one nonzero element and therefore taking into consideration the model indeterminacies. $\left\| \cdot \right\|$ denotes the usual Frobenius norm, and thus this index takes values between 0 and 1, where zero corresponds to perfect separation.

Tables 1, 2 and 3 show the average MD indices for sample sizes 1,000, 2,000 and 10,000 based on 2,000 repetitions. For space reasons, NSS-SOBI-gFOBI-1, NSS-SOBI-gFOBI-2 and NSS-SOBI-gFOBI-3 are denoted in the tables COMB-1, COMB-2 and COMB-3, respectively. For the simulations, the R packages JADE (Miettinen et al. 2016), tsBSS (Matilainen et al. 2019), fGarch (Wuertz et al. 2019), stochvol (Kastner 2016) and lattice (Sarkar 2008) were used within R 3.6.1 (R Core Team 2019).

The results confirm the above expectations. In the LP processes, SOBI is optimal, and the next best methods are the newly suggested combinations where the fourth-order cross-moment matrices are down-weighted. As soon as there is useful information available for the separation in these matrices, as in the SV and LP & SV scenarios, the combination methods are clearly superior. So, if there is doubt about the nature of the underlying processes, the combination methods are a safe choice.

## Example

**Fig. 2:**
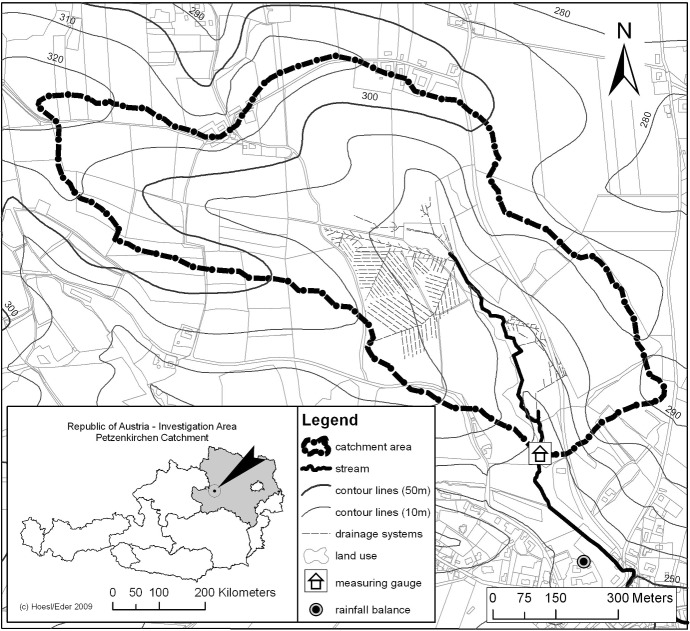
Petzenkirchen (Austria) catchment region. Reprinted with permission from Hoesl and Eder, appeared first in Eder et al. (2010)

As an illustrative example, a time series of light absorbance coefficients in water taken at a small stream near Petzenkirchen in Lower Austria is considered. The catchment region of the stream is shown in Fig. 2. The data were collected every 10 min from January 14, 2014, 2:20 p.m., until December 31, 2014, 11:50 p.m. Light at wavelengths of 200 nm, 202.5 nm, ..., 597.5 nm, 600 nm was emitted in a device through which stream water was channeled, and the amount of light absorbed was recorded. Depending on the water quality and on the possible presence of organic matter in the water, different wavelengths are absorbed to a varying degree. The absolute absorbance values are not of importance, only the relations of these values among the wavelengths under consideration. This means that the CTS is of length $T=50{,}620$ with dimension $p=161$. However, due to maintenance breaks and other problems such as measurement errors leading to nonpositive values, only 42,784 time points remain after some data cleaning and manipulation. A detailed description of the data and the background is available in Fischer (2020).

**Fig. 3:**
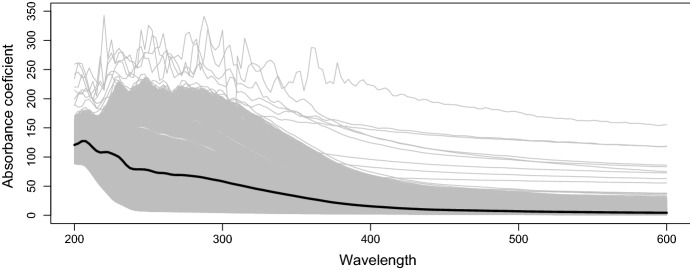
A selection of 2,000 observed absorption curves together with the mean curve

**Fig. 4:**
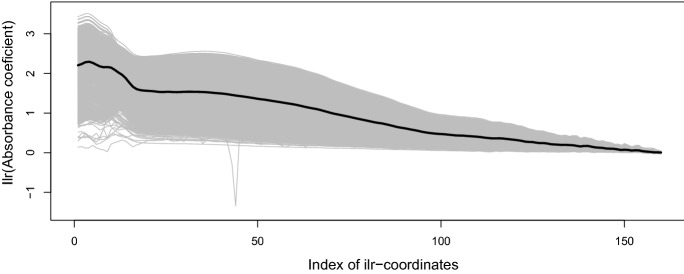
A selection of 2,000 absorption curves expressed in ilr coordinates, together with the mean curve

**Fig. 5:**
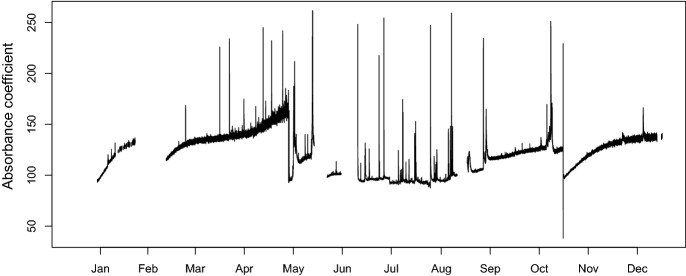
Absorption values at a wavelength of 200 nm over the study period

**Fig. 6:**
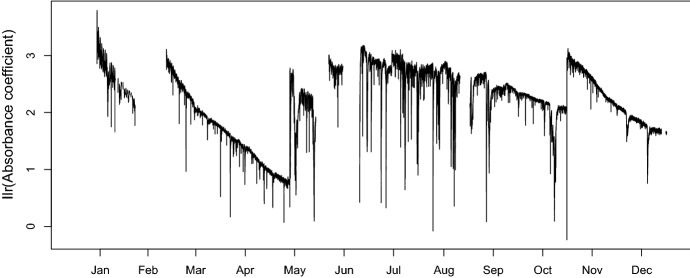
The first ilr component over the study period

Figure 3 shows a random selection of 2,000 absorption coefficient curves together with the mean curve in the original scale, while Fig. 4 shows the same observations expressed in ilr coordinates. The time series of the absorption coefficient at a wavelength of 200 nm is shown in Fig. 5, and the first ilr component in Fig. 6.

**Fig. 7:**
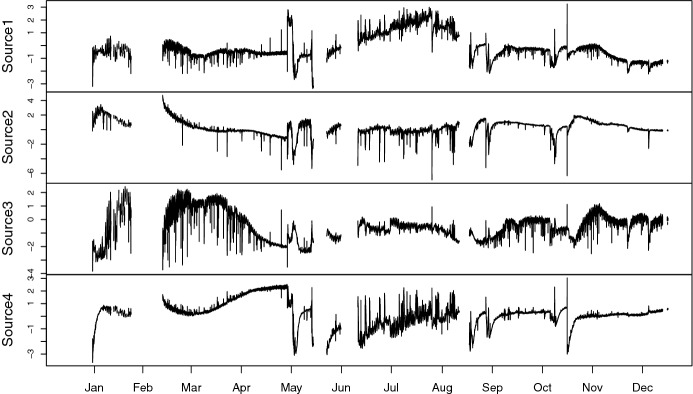
Estimated sources of NSS-SOBI-gFOBI-3

**Fig. 8:**
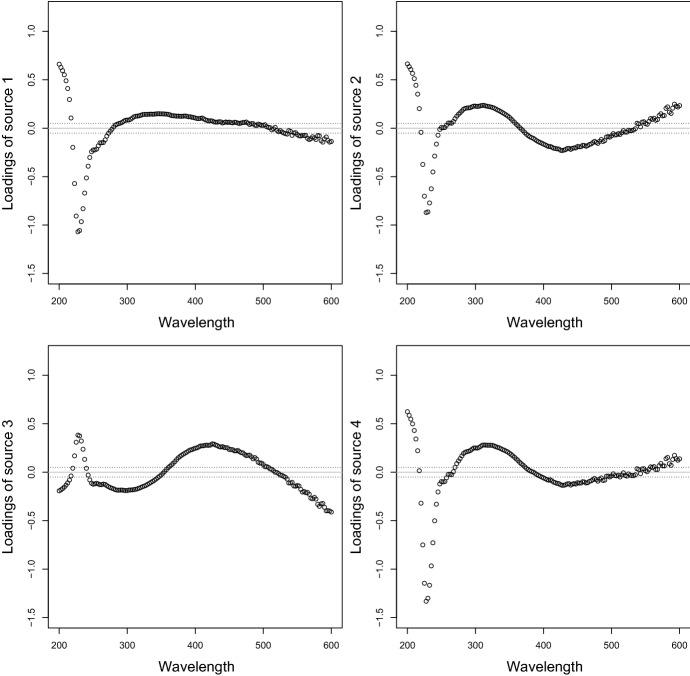
Clr loadings of NSS-SOBI-gFOBI-3 taking PCA into account

For analysis of the time series, the data are first expressed in ilr coordinates, and then principal component analysis (PCA) is performed. It turns out that four components together explain 99.91% of the variation. Thus, as the complete data are close to singularity, BSS is used with only these first four principal components. To account for the missing time points when performing BSS, only those pairs of observations are considered in the computation of the autocovariance matrices which correspond to the correct lag.

Figure 7 shows the recovered sources based on NSS-SOBI-gFOBI with weights $\alpha _j = 1/\max |{V_j}{[k,l]}|$, where the lags considered are ${\mathcal {T}}_1=\{6,12,\ldots ,72\}$, corresponding to hourly serial dependence from 1 to 12 h and ${\mathcal {T}}_2=\{78,84,\ldots ,144\}$. The second source could be seen as a general mean component, as most values are rather the same, while the first source emphasizes July and August, having the largest values there. Similarly, the third component has mostly large values from mid-January to April and could therefore be a spring component. The last component seems to focus on the transition from spring to summer in the months of April and May.

Figure 8 visualizes the clr loadings when also taking the PCA transformation into account. All four loading curves show the weights given to different wavelengths, referring to the different time periods of the four sources. The wavelength range around 220 nm seems to be the most important, but there are also clear patterns which may be interpreted by a subject matter expert. The loadings of source 3 are somewhat different from the other sources, and they could be related to the impact from farming, which would typically be visible during of March and April.

Note that most other BSS methods described above were also applied to the data, as well as other lag sets. In general, the components and loadings were fairly stable for all considered methods, and thus these results are not shown here. This can be seen as an indication of meaningful latent components which all contain features needed for the different methods to be able to recover them.

## Conclusions

CTS appear in many different contexts. In this paper it was shown how to perform BSS for CTS. BSS is well established for time series with different methods for different time series models. Usually, subject knowledge is available to guide the selection of the appropriate BSS methods, but there are methods available which cover several different models. In this spirit, a new combined method has been introduced here which yielded good results in simulation studies, and thus it was also applied on an environmental time series. BSS is often considered useful for multivariate time series, as it may help in modeling, prediction, dimension reduction and interpretation. The results here are in line with the findings published in Nordhausen et al. (2015); Bachoc et al. (2019), where spatial BSS methods proved useful for spatial compositional data.
